# Integrated trophic position decreases in more diverse communities of stream food webs

**DOI:** 10.1038/s41598-017-02155-8

**Published:** 2017-05-18

**Authors:** Naoto F. Ishikawa, Yoshito Chikaraishi, Naohiko Ohkouchi, Aya R. Murakami, Ichiro Tayasu, Hiroyuki Togashi, Jun-ichi Okano, Yoichiro Sakai, Tomoya Iwata, Michio Kondoh, Noboru Okuda

**Affiliations:** 1 Japan Agency for Marine-Earth Science and Technology, 2-15 Natsushima-cho, Yokosuka, Kanagawa 237-0061 Japan; 2 Institute of Low Temperature Science, Hokkaido University, Kita 19, Nishi 8, Kita-ku Sapporo, 060-0819 Japan; 3 Center for Ecological Research, Kyoto University, 2-509-3 Hirano, Otsu Shiga, 520-2113 Japan; 4 Research Institute for Humanity and Nature, 457-4 Motoyama, Kamigamo, Kita-ku Kyoto, 603-8047 Japan; 5 Tohoku National Fisheries Research Institute, Japan Fisheries Research and Education Agency, 3-27-5, Shinhama-cho, Shiogama, Miyagi 985-0001 Japan; 6 Lake Biwa Environmental Research Institute, 5-34 Yanagasaki, Otsu Shiga, 520-0022 Japan; 7 University of Yamanashi, 4-3-11 Takeda, Kofu Yamanashi, 400-8511 Japan; 8 Department of Environmental Solution Technology, Ryukoku University, 1-5 Yokoya, Seta Oe-cho, Otsu Shiga, 520-2194 Japan; 9 ETH Zürich, Sonneggstrasse, 5 8092 Zürich, Switzerland

## Abstract

The relationship between biodiversity and ecosystem functioning is an important theme in environmental sciences. We propose a new index for configuration of the biomass pyramid in an ecosystem, named integrated trophic position (iTP). The iTP is defined as a sum of trophic positions (i.e. the average number of steps involved in biomass transfer) of all the animals in a food web integrated by their individual biomass. The observed iTP for stream macroinvertebrates ranged from 2.39 to 2.79 and was negatively correlated with the species density and the Shannon–Wiener diversity index of the local community. The results indicate a lower efficiency of biomass transfer in more diverse communities, which may be explained by the variance in edibility hypothesis and/or the trophic omnivory hypothesis. We found a negative effect of biodiversity on ecosystem functioning.

## Introduction

The transfer of biomass via prey-predator interactions in food webs is a fundamental ecosystem process^1^. A higher efficiency of trophic transfer implies that more agricultural, livestock, and marine animal products are available per unit basal production, and such ecosystem functioning and service are critical for humanity^2^. As both the theoretical and empirical evidence suggest that primary productivity is sensitive to primary producers’ biodiversity^3–5^, the amount of biomass transferred across food webs is also likely to be influenced by the diversity of preys and predators^6, 7^. Therefore, a rigorous assessment of the relationship between biodiversity and the trophic transfer of biomass in ecosystems is a key challenge for predicting one of the consequences of ongoing species loss on the Earth^8^.

The trophic position (TP) is useful for integrating the flow of biomass from basal resources to an organism because the TP value reflects the average number of steps involved in biomass transfer^9^. Moreover, determining the mean TP for an entire animal community is indispensable for assessing conservation strategies, resource availability in fisheries, and the bioaccumulation of toxic substances^10, 11^. For example, the Marine Trophic Index (MTI), the mean TP of fisheries landings, has been used to evaluate the integrity of marine ecosystems^12, 13^, demonstrating that the average TP for commercially valuable fish has decreased globally since the 1970s. However, because the MTI did not consider non-fisheries species^14^, the index was biased toward commercially important and large predatory fish. Thus, it appears that the MTI was unable to represent the biomass flow in a whole community. Until recently, few studies have holistically examined the mean TPs of all animals in food webs, with the exception of a study by Williams & Martinez (2004), which reconstructed a trophic network to estimate the average TP for a whole community by using a large dataset based on gut contents^15^.

In the present study, we propose a new index of food web properties, the integrated trophic position (iTP), which we defined as the summed TPs of all animals in a food web integrated by their individual biomass. The iTP of an animal community containing *n* species is represented as:

1 $$iTP=\sum _{i=1}^{n}(T{P}_{i}\times \frac{{B}_{i}}{{B}_{T}})$$

where *TP*_*i*_ is the TP of species *i*, and *B*_*i*_ and *B*_*T*_ are the biomass of species *i* and the total biomass of the focal community per unit area or space, respectively. Hence, the iTP can be a measure for the average number of times *x* that the assimilated organic matter is transferred along trophic pathways in a food web (i.e. iTP = *x* + 1). It should be noted that the iTP for macroinvertebrates in the present study does not include primary producers (i.e. TP = 1) and predatory vertebrates (e.g. fish).

The iTP can be easily determined by measuring the TP of the composite biomass in the community. This is a unique and advantageous feature because it is practically infeasible to calculate the iTP by integrating the TP and biomass for each species in an ecosystem, which are measured individually for each of the animals included. The TP has often been estimated using stable nitrogen isotope ratios (*δ* ^15^N) based on the bulk tissue of an animal and its food source(s) according to a constant enrichment of ^15^N during each trophic transfer, although fluctuations in the *δ* ^15^N values of basal resources often lead to high uncertainty in TP estimates^9, 16, 17^. More recently, the compound-specific isotope analysis of amino acids (CSIA-AA) has emerged as a new analytical technique for estimating the TPs of animals more precisely and accurately in diverse ecosystems^18–21^. The CSIA-AA method only needs the *δ* ^15^N value for a focal animal and no source data, so it is independent of the *δ* ^15^N fluctuations in basal resources, thereby leading to much smaller errors in the TP estimates compared to bulk *δ* ^15^N analysis^22^.

To explore the effects of biodiversity loss on organic matter flows in ecosystems, we focused on benthic macroinvertebrate communities in stream ecosystems where the spatial scale of the food webs can be constrained using a simple quadrat sampling methodology^23^. The community composition often differs greatly among stream reaches, even within a watershed, depending on multiple factors such as the longitudinal heterogeneity of surrounding landscapes, disturbance regime, and anthropogenic impacts^24^. These contrasting features are expected to contribute to variations in the iTP values of local macroinvertebrate food webs. A previous CSIA-AA study^25^ showed that macroinvertebrate individuals in stream food webs had TP values ranging between 2 and 3, but the iTP was not investigated for each community. In the present study, macroinvertebrate communities were collected from 15 sites in the Yasu River, Japan, to estimate the iTP for each local community using CSIA-AA, where their spatial variation was examined relative to the community properties (e.g. diversity indices) of food webs.

## Results

**Figure 1:**
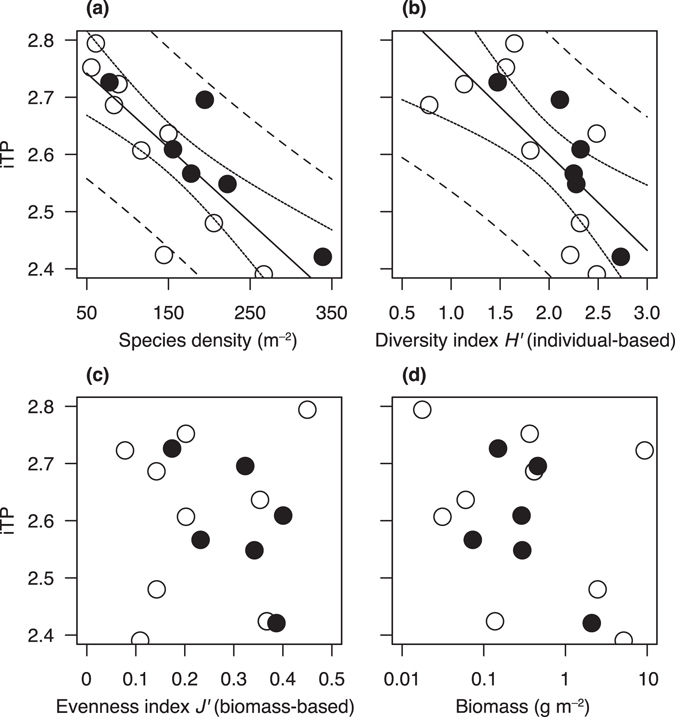
Relationships between integrated trophic position (iTP) for macroinvertebrates and (**a**) species density (m^−2^), (**b**) the individual-based Shannon–Wiener diversity index (*H*′), (**c**) the biomass-based Pielou evenness index (*J*′), and (**d**) total biomass (g m^−2^). Open circles indicate stream orders 1 or 2, and shaded circles indicate stream orders 3 or 4. Solid, dotted, and dashed lines in (**a**) and (**b**) represent the regression equation, 95% confidence interval, and 95% prediction interval, respectively.

For each local community, the biomass-integrated *δ* ^15^N values for glutamic acid (*δ* ^15^N_Glu_) and phenylalanine (*δ* ^15^N_Phe_) ranged from 12.0 to 21.7‰ and from –3.7 to 7.0‰, respectively (Table 1). The *δ* ^15^N_Glu_ and *δ* ^15^N_Phe_ values were significantly correlated with each other (*R* ^*2*^ = 0.89, *p* < 0.001). The observed iTP for macroinvertebrates ranged from 2.39 to 2.79 (Table 1), and had negative relationships with species density (*R* ^*2*^ = 0.66, *p* < 0.001) and individual-based diversity index *H*′ (*R* ^*2*^ = 0.51, *p* = 0.003) (Fig. 1a and b). The species density tended to increase with the total number of individuals found in a unit area (Fig. S2a) because a higher population density increases the probability of sampling a larger number of species in a given area^26^. The effects of diversity indices (i.e. both the species density and *H*′) on the iTP were significant, indicating that the species diversity was primarily responsible for the variation in the iTP. The biomass-based *J*′ (i.e. evenness index) and biomass itself were not significantly correlated to the iTP (*R* ^*2*^ < 0.01, *p* = 0.67–0.95, Fig. 1c and d

**Table 1:** Dataset analysed in the present study.

Site #	Latitude	Longitude	Stream order	Species density (m^−2^)	Total biomass (g m^−2^)	Shannon–Wiener index (*H*′)	Pielou index (*J*′)	*δ* ^15^N_Glu_ (‰)	*δ* ^15^N_Phe_ (‰)	iTP
1	34°58′57″N	136°07′49″E	2	267	5.16	2.49	0.10	16.8	2.8	2.39
2	34°59′09″N	136°06′45″E	3	339	2.10	2.73	0.37	14.5	0.3	2.42
3	35°02′33″N	136°03′47″E	2	144	0.14	2.21	0.36	12.0	−2.2	2.42
4	35°01′08″N	136°02′57″E	1	206	2.47	2.31	0.14	21.7	7.0	2.48
5	34°54′06″N	136°12′36″E	3	222	0.30	2.28	0.31	17.4	2.3	2.55
6	34°56′58″N	136°18′46″E	3	178	0.07	2.25	0.29	12.6	−2.7	2.57
7	35°00′29″N	136°23′51″E	1	117	0.03	1.81	0.20	16.2	0.6	2.61
8	34°56′30″N	136°16′15″E	4	156	0.29	2.32	0.39	16.8	1.2	2.61
9	34°55′52″N	136°08′51″E	2	150	0.06	2.48	0.34	12.1	−3.7	2.64
10	34°57′02″N	136°10′48″E	2	83	0.41	0.78	0.09	17.6	1.4	2.69
11	34°57′26″N	136°20′17″E	3	194	0.46	2.11	0.32	16.7	0.4	2.70
12	35°01′09″N	136°02′58″E	1	89	9.31	1.13	0.08	20.5	4.0	2.72
13	34°55′33″N	136°09′53″E	3	78	0.15	1.47	0.14	18.9	2.4	2.73
14	34°53′21″N	136°16′42″E	1	56	0.36	1.56	0.20	17.4	0.6	2.75
15	34°59′43″N	136°23′20″E	2	61	0.02	1.64	0.45	19.4	2.3	2.79

Species density, total biomass, the individual-based Shannon–Wiener diversity index (*H*′), and the biomass-based Pielou evenness index (*J*′) were each calculated by averaging the values from two sampling replications at each site.

).

It is possible that the iTP was affected by the TPs of a few dominant species in terms of biomass in the local community because the iTP value is determined by all of the macroinvertebrate individuals. If a few large carnivores or large algivores dominated the local communities, iTP would have a negative or positive correlation, respectively, with *J*′. A low *J*′ and a moderate iTP would be expected if the community was dominated by both carnivores and algivores, or by a few species with moderate TPs. However, the biomass-based evenness index *J*′ was not correlated to the iTP, and thus the biomass-integrated TP was not attributable to the skewness of the biomass distribution among species. In case where production and biomass are interchangeable, and trophic transfer efficiency of biomass is identical among communities, theory expects a positive relationship between biomass and the iTP. This is because higher productivity (i.e. a larger amount of biomass) can support a longer food chain length (FCL)^27^, which is comparable to a higher iTP. However, our results indicate that the trophic transfer efficiency of biomass is different among local communities.

## Discussion

**Figure 2:**
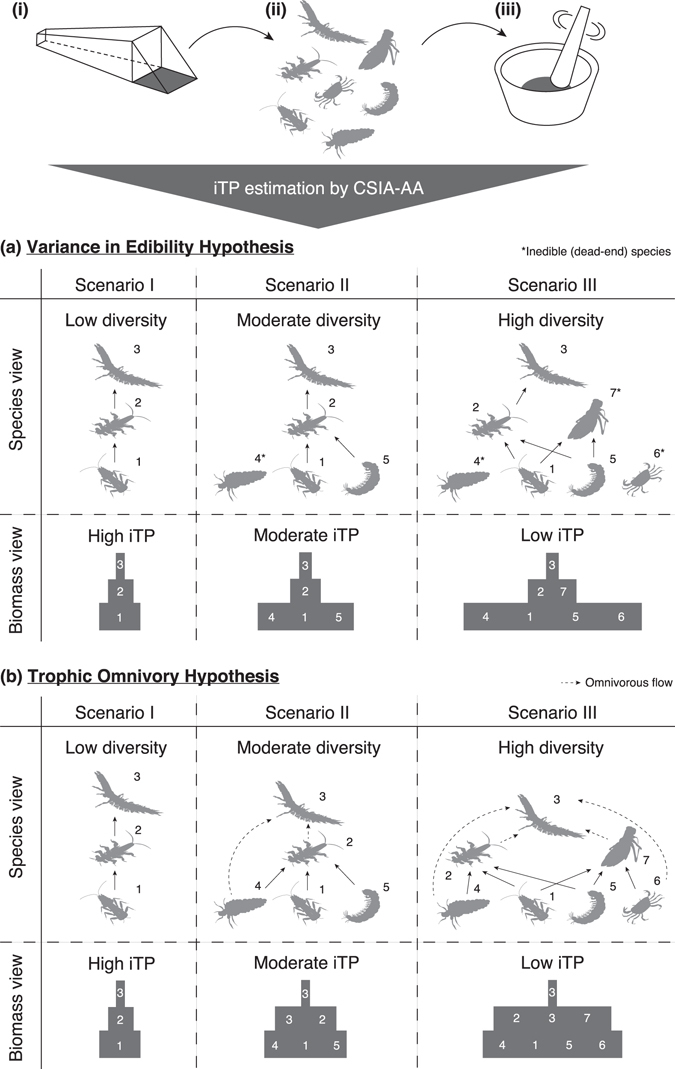
Schematic overview of the present study: (**i**) sample collection from a given area; (**ii**) sorting animal individuals; (**iii**) homogenizing a whole community followed by CSIA-AA; and a simplified illustration of potential hypotheses. (**a**) Variance in edibility hypothesis and/or (**b**) Trophic omnivory hypothesis might explain the negative relationship between biodiversity and the iTP for macroinvertebrates. Numbers are used for species identification in the text. The asterisk and dashed arrow denote inedible (dead-end) species and omnivorous flow, respectively. See the text for details.

A lower iTP value with a higher species diversity in macroinvertebrates implies that smaller proportions of the biomass were assimilated by predators from more diverse communities. In natural food webs, a more diverse prey community is known to increase the likelihood of including species with resistance to predation (e.g. a hard shell or toxicity)^7, 28, 29^. In these cases, the predation-resistant “inedible” species (e.g. species 4, 6 and 7 in Fig. 2a) compete with edible species for common resources, thereby decreasing the biomass flow from edible prey to a predator (Fig. 2a, Scenarios II and III). McNeely *et al*.^30^ showed that removal of an armoured and predation-resistant grazer, the larvae of the caddisfly *Glossosoma penitum*, increased the biomass of competitive vulnerable grazers and slightly increased the biomass of predators^30^. However, inedible prey species are unlikely to be included in less-diverse communities where only a few species dominate the faunal components, thereby increasing the amount of biomass transferred through trophic pathways (Fig. 2a, Scenario I). Moreover, as shown in Scenario III in Fig. 2a, if an inedible predator (species 7) competes with the other edible predator (species 2) for their common preys (e.g. species 1), the biomass flow from species 1 to 2 is indirectly reduced via the top-down effect of species 7 on species 1^31^. This may in turn decrease the flow from species 2 to species 3 (Fig. 2a, Scenario III).

Another, but not mutually exclusive, mechanism is a potential increase of trophic omnivory in communities including more prey species, where a predator eats preys from more than one TP^7, 32^. In Fig. 2b, we assumed that the top predator (species 3) eats intermediate predators (species 2 and 7) as well as their respective preys (species 4 and 6). In this case, the biomass that is transferred via species 3’s direct feeding on species 4 and 6 is allocated at the intermediate level of the biomass pyramid instead of the top level (Fig. 2b, Scenarios II and III). Furthermore, it reduces trophic flows of the biomass from species 4 and 6 to species 2 and 7, respectively, if total flow from a prey to its predators is constant. This may finally reduce the flow from species 2 and 7 to species 3, which decreases the biomass at the top level of the pyramid. Therefore, food webs with the trophic omnivory are expected to show lower iTP values than those without. Overall, in diverse communities, a major increase of the biomass at the bottom and intermediate levels of the pyramid is predicted by the variance in edibility and the trophic omnivory hypotheses, respectively. Either or both of the two hypotheses can be useful for explaining a negative correlation between species diversity and iTP.

The spatial variation in the iTP for macroinvertebrates may be attributed to resource subsidy from terrestrial ecosystems (e.g. inputs of amino acids derived from terrestrial vascular plants). In Eq. 5, the β value is −3.4‰ for the aquatic food chain, but +8.4‰ for the terrestrial food chain^22, 33^. Thus, if terrestrial subsidization makes a great contribution to the biomass of the macroinvertebrate community, its iTP value would be underestimated. A previous study in the Yasu River estimated the relative contribution of terrestrial resources to macroinvertebrate communities as 10%–30%^25^, which is equal to variations of 0.16–0.47 units in TP estimates. However, when we analysed only the middle and downstream study sites (i.e. third or fourth stream orders), where macroinvertebrate communities may depend greatly on autochthonous algal production, the significant negative relationships between the iTP and both species density and *H*′ were still evident (*N* = 6, *R* ^*2*^ = 0.78, *p* = 0.02, Fig. 1a and b). Therefore, terrestrial subsidy was unlikely to be a major factor that determined the pattern found in the present study.

The mechanisms that underlie the negative relationship between the iTP for macroinvertebrates and species diversity are still unclear. In extremely simple communities with no predators (e.g. only plants and herbivores), increasing diversity should add an additional trophic level, which in turn increases the iTP. Thus, a positive relationship between iTP and species diversity might be found if the species density is much less than 50 m^−2^ (Fig. 1a). When the species density increases to >50 m^−2^, the iTP is decreased by inedible prey (the variance in edibility hypothesis) or edible prey (the trophic omnivory hypothesis) for species both at intermediate and high trophic levels. The addition of edible prey for species only at an intermediate trophic level (e.g. species 5 in Fig. 2) is unlikely to affect the iTP, and this is probably why >100 species are required per m^2^ to decrease iTP by at most 0.2 units.

Fish and amphibian species were not investigated in the present study. According to a general rule that consumers are more mobile with increasing TP^34^, the spatial scale of the fish and amphibian movement is much larger than that of macroinvertebrates, which made it difficult to involve these animals in estimating the iTP of a local food web in our study system. Furthermore, since the present study was intensively conducted in a single watershed and over a single season, further studies should assess spatiotemporal variations in the iTP by comparing multiple watersheds and seasons.

Despite several limitations, the iTP index is comparable to traditional food web properties such as the FCL. The mean FCL has been used as a quantitative measure for the average TP among all top predators in each of the respective food chains nested in a focal food web^27, 35, 36^. Interestingly, a meta-analysis by Doi *et al*. (2012) found that the FCL in lakes tended to decrease as the number of endemic species increased^37^, and this pattern is similar to our identification of a negative relationship between species richness and iTP in the stream macroinvertebrate community. The traditional FCL is highly sensitive to the sampling procedure and typically focuses only on top predators, so it does not consider the “dead-end” species within trophic networks, except for the top predators themselves. By contrast, the iTP is highly independent of sampling bias and considers the effects of inedible species in its estimate, which are never transferred to the top predators. For example, the FCL in Fig. 2a (i.e. TP of the top predator species 3) does not change, regardless of the species diversity, whereas that in Fig. 2b decreases with decreasing the iTP in more diverse communities. In other words, under the variance in edibility hypothesis, only the iTP captures changes in the top predator’s biomass per unit basal production (i.e. trophic transfer efficiency), which is a “multi-trophic” ecosystem functioning^7^. It should be noted that neither FCL nor iTP detect changes in the trophic transfer efficiency under the trophic omnivory hypothesis. Our results are supported by a few previous studies that showed negative relationships between prey diversity and trophic transfer efficiency^38, 39^, and contrasts with most of the previous understanding of the positive relationship between biodiversity and ecosystem functioning without trophic interactions. In conclusion, the iTP index provides a novel, simple, and universal measure of food web properties, such as the trophic transfer efficiency and the configuration of the biomass pyramid in an ecosystem. It will be applicable to a wide range of taxonomic groups inhabiting a certain area or space in various ecosystems and will improve our understanding of the relationships between biodiversity (i.e. species richness) and ecosystem functioning (i.e. trophic transfer of biomass).

## Methods

### Study sites and sample collection

The Yasu River is the largest tributary of Lake Biwa in Japan. The headwater streams of the Yasu River run through forested mountainous areas, whereas the middle and downstream rivers are influenced by urban development and agriculture. The nitrate concentrations and isotope values increase longitudinally from upstream to downstream in the Yasu River due to anthropogenic nitrogen loading from human-dominated terrestrial environments^40^. In total, 15 sites were established within the watershed, including streams of different sizes (first to fourth order streams with catchment areas ranging from 0.3 to 71 km^2^) and different surrounding landscapes.

In October 2012, three samples of stream macroinvertebrates including Annelida, Arthropoda, Mollusca, Nematomorpha, and Platyhelminthes were collected from each study site using a Surber net (30 cm × 30 cm area, mesh size = 475 µm). In the laboratory, two of the three samples were fixed with formalin to identify the lowest recognizable taxa of macroinvertebrates using a binocular microscope (Table S1). Because 124 taxa out of 130 in total (95%) were identified to species level, we regarded taxonomic diversity as species diversity in the present study. The number of individuals for each species was counted (Tables S2 and S3). Individuals were then selected from each species to measure individual biomass by average wet weight. For molluscs, muscles were used to represent their biomass. The biomass values of armoured caddisflies were measured without cases. The biomass of each species was calculated as the average individual biomass multiplied by the number of individuals.

Using the number of individuals and biomass of each species and species density (i.e. the number of species), the Shannon–Wiener diversity index (*H*′) was determined based on either the number of individuals (i.e. individual-based) or biomass (i.e. biomass-based) for each sample as:

2 $${H}^{^{\prime} }=-\sum _{i=1}^{S}({p}_{i}\times ln{p}_{i})$$

where *S* is the total number of species in the community, and *p*_*i*_ is the proportional number of individuals or the biomass of species *i* relative to the local community (0 ≤ *p*_*i*_ ≤ 1). When *p*_*i*_ was equal among all species, *H*′ took the maximum value as follows:

3 $${H^{\prime} }_{{\rm{\max }}}=-\sum _{i=1}^{S}(\frac{1}{S}\times \,\mathrm{ln}\,\frac{1}{S})=-\,S\times (\frac{1}{S}\times \,\mathrm{ln}\,\frac{1}{S})=\,\mathrm{ln}\,S$$

Therefore, the individual-based and biomass-based evenness indices (Pielou *J*′) (0 ≤ *J*′ ≤ 1) were derived as follows:

4 $${J}^{^{\prime} }=\frac{{H}^{^{\prime} }}{{{H}^{^{\prime} }}_{max}}=\frac{{H}^{^{\prime} }}{{\rm{l}}{\rm{n}}S}$$

The values of the species density (m^−2^), *H*′, and *J*′ for each of the two sampling replications were averaged for each site. For the remaining sampling replication, all of the macroinvertebrates were manually selected, dried in an oven at 60 °C for 24 h, blended, homogenized, and weighed to calculate the community biomass (g m^−2^). For the shell organisms, only soft tissues contributed to the community biomass. The homogenized macroinvertebrate samples were then used for the isotope analysis described below.

### Isotope measurements and iTP calculations

For CSIA-AA, amino acids in the homogenized sample were purified by HCl hydrolysis followed by *N*-pivaloyl/isopropyl (Pv/iPr) addition, according to the improved procedures described by Chikaraishi *et al*. (2007)^19^. The *δ* ^15^N values of amino acids were determined using the modified method described by Chikaraishi *et al*. (2010)^41^, using a Delta XP plus isotope ratio mass spectrometer (Thermo Fisher Scientific, U.S.A.) coupled to a gas chromatograph (GC6890N; Agilent Technologies, U.S.A.) via combustion and reduction furnaces. Isotopic reference mixtures of nine amino acids (*δ* ^15^N ranging from −26.5 to +46.3‰) were analysed every five or six sample to confirm the reproducibility of the isotope measurements. Analytical errors (1 σ) for the standards were better than 0.5‰ with a minimum sample quantity of 0.5 nmol N.

During amino acid metabolism, glutamic acid is subject to deamination, leading to an increase in the *δ* ^15^N value (~8.0‰) associated with trophic transfer. Conversely, phenylalanine retains its amino group because of the low deamination flux during metabolism, thereby resulting in a negligible increase in the *δ* ^15^N value (~0.4‰) during trophic transfer. Based on this principle, the *δ* ^15^N values of glutamic acid and phenylalanine within a single animal were employed to determine its TP using the following equation with analytical precision (1 σ) of 0.12 units^22^:

5 $$TP=\frac{{\delta }^{15}{{\rm{N}}}_{{\rm{G}}{\rm{l}}{\rm{u}}} - {\delta }^{15}{{\rm{N}}}_{{\rm{P}}{\rm{h}}{\rm{e}}}+\beta }{TDF}+1$$

where *δ* ^15^N_Glu_ and *δ* ^15^N_Phe_ are the *δ* ^15^N values for glutamic acid and phenylalanine, respectively. β is a constant value for the difference between *δ* ^15^N_Phe_ and *δ* ^15^N_Glu_ in a primary producer at the base of a food chain (i.e. −3.4 for algae and cyanobacteria and +8.4 for vascular plants)^22, 35^. A β value of −3.4 was used in our stream food webs, where benthic algae were regarded as primary producers. TDF is the trophic discrimination factor between *δ* ^15^N_Glu_ and *δ* ^15^N_Phe_ during each trophic transfer (i.e. 8.0‰–0.4‰ = 7.6‰). The iTP for each macroinvertebrate community was estimated as the TP value in the homogenized, biomass-integrated macroinvertebrate sample, which was determined using the *δ* ^15^N_Glu_ and *δ* ^15^N_Phe_ values and Eq. 5.

### Statistical analysis

Regression analyses were performed to test correlations between iTP and the four parameters (i.e. the species density, the Shannon–Wiener diversity index *H*′, the Pielou evenness index *J*′, and the biomass). The significance level was set at α = 0.05.

## Electronic supplementary material

Supplementary Information
