# Supplementary material for: Integrated trophic position decreases in more diverse communities of stream food webs
Source: Sci Rep. 2017 May 18;7:2130. doi: 10.1038/s41598-017-02155-8 (PMC5437047; doi:10.1038/s41598-017-02155-8)
Supplement: Supplementary file 1 — Supplementary Information [file 41598_2017_2155_MOESM1_ESM.pdf]

Supplementary Information

Integrated trophic position decreases in more diverse communities of stream food webs

Naoto F. Ishikawa[1,*,†], Yoshito Chikaraishi[1,2], Naohiko Ohkouchi[1], Aya R. Murakami[3], Ichiro Tayasu[4], Hiroyuki Togashi[5], Jun-ichi Okano[3], Yoichiro Sakai[6], Tomoya Iwata[7], Michio Kondoh[8], Noboru Okuda[4]

[1]Japan Agency for Marine-Earth Science and Technology, 2-15 Natsushima-cho, Yokosuka, Kanagawa 237-0061, Japan

[2]Institute of Low Temperature Science, Hokkaido University, Kita 19, Nishi 8, Kita-ku, Sapporo, 060-0819 Japan

[3]Center for Ecological Research, Kyoto University, Hirano 2-509-3, Otsu, Shiga 520-2113, Japan

[4]Research Institute for Humanity and Nature, 457-4 Motoyama, Kamigamo, Kita-ku, Kyoto, 603-8047 Japan

[5]Tohoku National Fisheries Research Institute, Japan Fisheries Research and Education Agency, 3-27-5, Shinhama-cho, Shiogama, Miyagi 985-0001, Japan

[6]Lake Biwa Environmental Research Institute, 5-34 Yanagasaki, Otsu, Shiga 520-0022, Japan

[7]University of Yamanashi, 4-3-11 Takeda, Kofu, Yamanashi 400-8511, Japan

[8]Department of Environmental Solution Technology, Ryukoku University, 1-5 Yokoya, Seta Oe-cho, Otsu, Shiga 520-2194, Japan

*Correspondence to: naoto.f.ishikawa@gmail.com

†Current address: ETH Zürich, Sonneggstrasse 5 8092 Zürich, Switzerland

Fig. S1. $\delta^{15}N_{Glu}$ (shaded square) and $\delta^{15}N_{Phe}$ (open square) values, and the iTP values (circle) calculated for macroinvertebrate communities collected from 15 sites on the Yasu River. Open circles indicate stream orders 1 or 2, and shaded circles indicate stream orders 3 or 4.

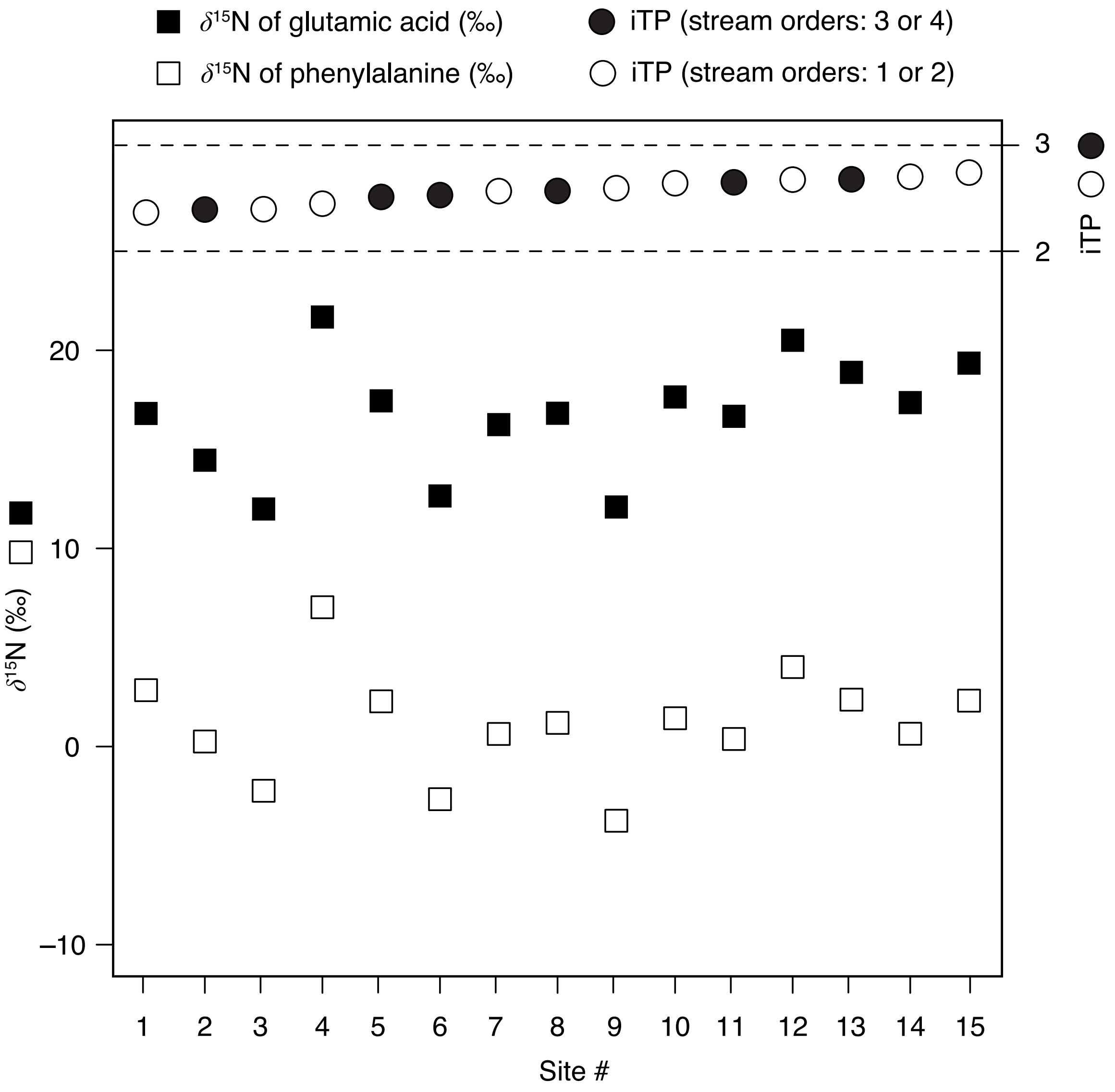

Fig. S2. Relationships of (**a**) species density (m^{-2}) or (**b**) iTP to the number of individuals (m^{-2}). The enlarged right panels exclude one sample with an extremely large number of individuals (i.e. site 10). Relationships between the iTP and (**c**) biomass-based Shannon–Wiener index (*H′*) or (**d**) individual-based Pielou evenness index (*J′*). Open circles indicate stream orders 1 or 2, and shaded circles indicate stream orders 3 or 4.

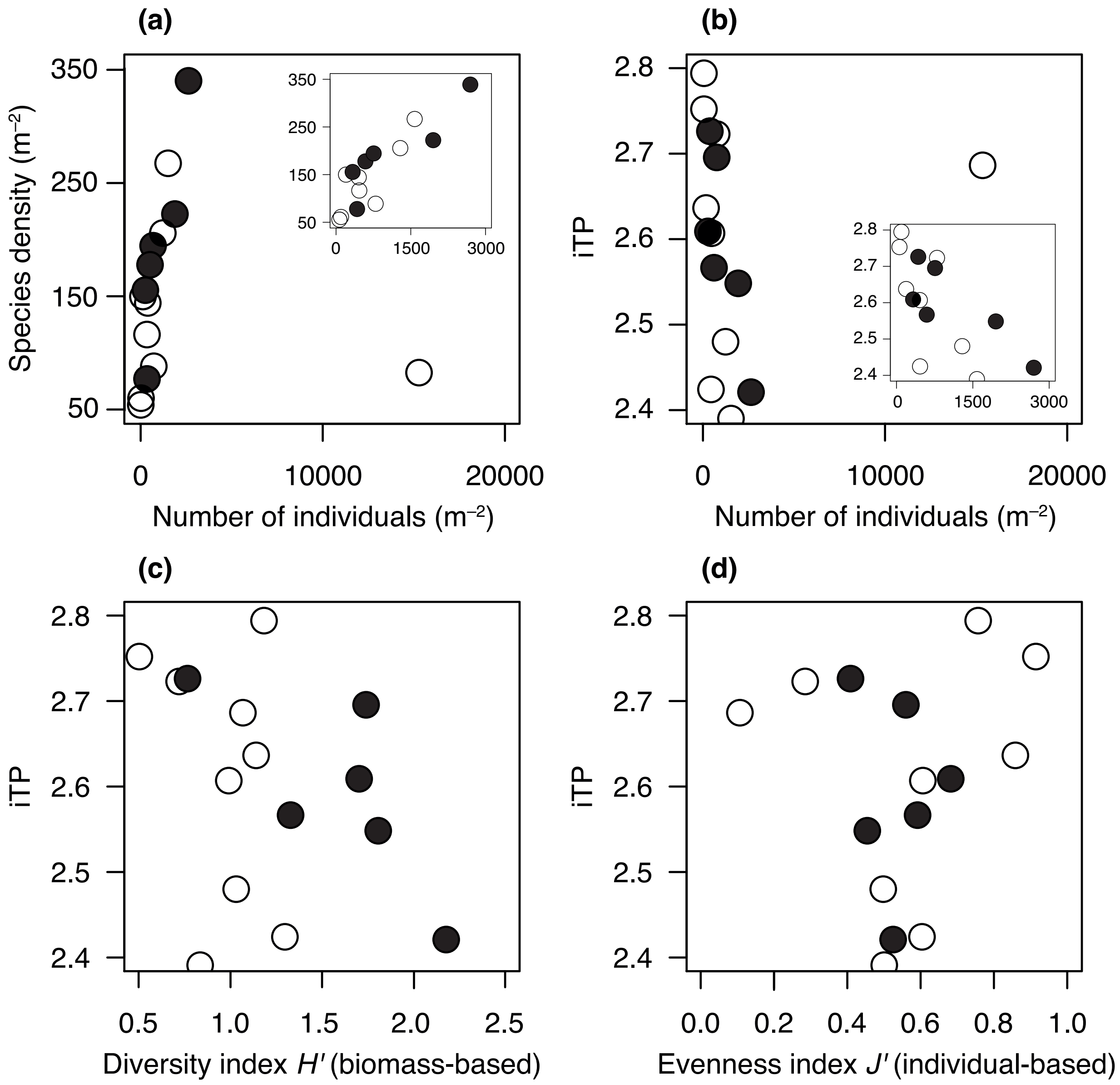

Fig. S3. Relationships between integrated trophic position (iTP) for macroinvertebrates and species density (m^{-2}) and the individual-based Shannon–Wiener diversity index (*H'*) for the first replication (**a** and **b**, respectively) and those for the second replication (**c** and **d**, respectively). All correlations were significant (R^2 = 0.44-0.68, p < 0.01). Solid, dotted, and dashed lines represent the regression equation, 95% confidence interval, and 95% prediction interval, respectively.

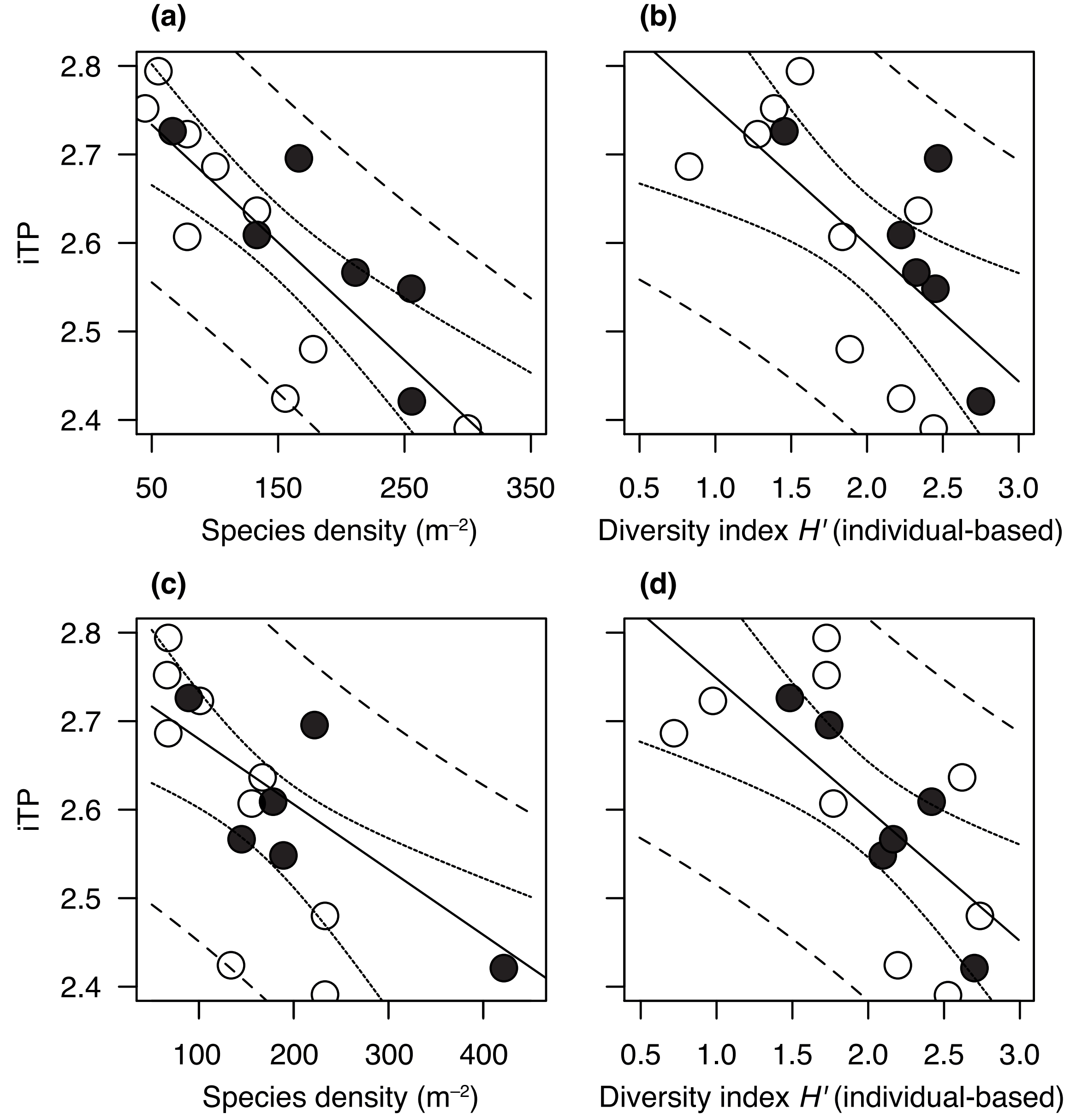

Table S1. Taxonomic information. Individual biomass (mg) was estimated by weighing several individuals from each taxon.

Taxon #	Name of order	Name of family	Name of species	Individual biomass	Individual biomass SD
1	Tricladida	Dugesiidae	*Dugesia japonica*	0.9	0.76
2	Gordioida	Chordodidae	*Chordodes* sp.	571.2	5.73
3	Architaenioglossa	Viviparidae	*Sinotaia quadrata histrica*	1385.6	135.62
4	Discopoda	Pleuroceridae	*Semisulcospira libertina*	44.6	36.82
5			*Semisulcospira reiniana*	180.0	172.55
6	Basommatophora	Lymnaeidae	*Fossaria ollula*	5.1	0.00
7		Physidae	*Physa acuta*	7.1	4.76
8	Veneroida	Corbiculidae	*Corbicula* sp.	117.0	89.14
9	Tubificida	Naididae	*Branchiura sowerbyi*	1.7	0.65
10			*Nais* sp.	0.6	0.66
11			Naididae gen. sp.	0.6	0.66
12			Naididae gen. spp.	0.6	0.66
13	Lumbricida	Megascolecidae	Megascolecidae gen. sp.	5.6	5.73
14	Arhynchobdellida	Erpobdellidae	*Erpobdella octoculata*	252.9	527.73
15			*Erpobdella testacea*	363.3	0.00
16	Amphipoda	Crangonyctidae	*Crangonyx floridanus*	1.0	0.60
17	Isopoda	Asellidae	*Asellus hilgendorfi hilgendorfi*	1.1	1.17
18	Decapoda	Atyidae	*Neocaridina denticulata*	57.6	0.00
19		Cambaridae	*Procambarus clarkii*	1689.7	3351.66
20		Potamidae	*Geothelphusa dehaani*	104.7	82.69

Table S1. Continued.

Taxon #	Name of order	Name of family	Name of species	Individual biomass	Individual biomass SD
21	Ephemeroptera	Baetidae	*Acentrella gnom*	0.4	0.08
22			*Alainites yoshinensis*	0.6	0.27
23			*Baetiella japonica*	1.2	1.10
24			*Baetis sahoensis*	2.0	0.00
25			*Baetis taiwanensis*	0.6	0.31
26			*Baetis thermicus*	0.9	0.86
27			*Baetis* sp. J	1.1	0.07
28			*Baetis* sp.	1.1	0.00
29			*Labiobaetis atrebatinus orientalis*	0.5	0.14
30			*Tenuibaetis flexifemora*	0.9	0.44
31		Heptageniidae	*Ecdyonurus yoshidae*	0.7	0.48
32			*Epeorus curvatulus*	7.4	6.92
33			*Epeorus* sp.	4.1	2.29
34		Isonychiidae	*Isonychia japonica*	5.3	8.87
35		Leptophlebiidae	*Choroterpes altioculus*	0.4	0.33
36			*Paraleptophlebia* sp.	0.1	0.05
37		Ephemeridae	*Ephemera japonica*	5.1	5.41
38			*Ephemera orientalis*	3.1	4.40
39			*Ephemera strigata*	5.7	3.02
40		Potamanthidae	*Potamanthus formosus*	0.7	0.55

Table S1. Continued.

Taxon #	Name of order	Name of family	Name of species	Individual biomass	Individual biomass SD
41		Ephemerellidae	*Cincticostella* sp.	0.3	0.08
42			*Ephemerella setigera*	0.3	0.00
43			*Torleya japonica*	0.7	1.07
44			*Uracanthella punctisetae*	0.2	0.05
45	Odonata	Calopterygidae	*Calopteryx atrata*	4.7	3.61
46		Gomphidae	*Davidius* sp.	0.4	0.00
47			*Nihonogomphus viridis*	220.9	246.07
48			*Onychogomphus viridicostus*	39.0	34.95
49		Cordulegasteridae	*Anotogaster sieboldii*	N/A	N/A
50	Plecoptera	Nemouridae	*Amphinemura* sp.	1.0	0.35
51			*Nemoura* sp.	0.2	0.08
52			*Protonemura* sp.	0.1	0.00
53		Chloroperlidae	Chloroperlidae gen. sp.	0.8	0.29
54		Perlidae	*Kamimuria* sp.	6.3	4.68
55			*Neoperla* sp.	3.4	2.19
56			*Oyamia* sp.	237.0	N/A
57			*Paragnetina* sp.	5.3	0.28
58			*Togoperla* sp.	40.6	0.00
59			Perlinae gen. sp.	0.2	0.05
60	Hemiptera	Gerridae	*Metrocoris histrio*	19.6	0.00

Table S1. Continued.

Taxon #	Name of order	Name of family	Name of species	Individual biomass	Individual biomass SD
61		Aphelochiridae	*Aphelocheirus vittatus*	3.8	5.00
62	Megaloptera	Corydalidae	*Protohermes grandis*	44.2	0.00
63	Neuroptera	Nevrorthidae	Nevrorthidae gen. sp.	1.9	0.00
64	Ttrichoptera	Hydropsychidae	*Cheumatopsyche brevilineata*	5.7	2.63
65			*Cheumatopsyche infascia*	0.5	0.17
66			*Cheumatopsyche* sp.	0.3	0.15
67			*Diplectrona* sp.	0.6	0.00
68			*Hydropsyche albicephala*	2.3	1.33
69			*Hydropsyche ancorapunctata*	11.6	0.00
70			*Hydropsyche orientalis*	5.0	2.64
71		Philopotamidae	*Dolophilodes* sp.	1.3	1.41
72		Stenopsychidae	*Stenopsyche marmorata*	22.0	37.24
73			*Stenopsyche sauteri*	39.5	19.76
74			*Stenopsyche* sp.	N/A	N/A
75		Glossosomatidae	*Agapetus* sp.	1.4	0.58
76			*Glossosoma* sp.	4.3	2.50
77			Glossosomatidae gen. spp.	0.1	0.00
78		Hydrobiosidae	*Apsilochorema sutshanum*	0.2	0.00
79		Rhyacophilidae	*Rhyacophila kawamurae*	0.8	0.95
80			*Rhyacophila lezeyi*	0.7	0.49

Table S1. Continued.

Taxon #	Name of order	Name of family	Name of species	Individual biomass	Individual biomass SD
81			*Rhyacophila shikotsuensis*	29.2	0.00
82			*Rhyacophila* sp.	6.8	9.48
83		Goeridae	*Goera japonica*	3.6	3.51
84			*Goera* sp.	2.9	4.23
85		Lepidostomatidae	*Lepidostoma* sp.	0.04	0.00
86		Leptoceridae	*Trichosetodes japonicus*	0.1	0.07
87		Sericostomatidae	*Gumaga orientalis*	0.8	0.70
88	Lepidoptera	Crambidae	*Potamomusa midas*	1.6	0.00
89	Diptera	Tipulidae	*Antocha* sp.	1.2	1.34
90			*Dicranota* sp.	3.1	2.17
91			*Hexatoma* sp.	5.9	6.68
92			*Tipula* sp.	253.1	0.00
93		Chironomidae	*Brillia* sp.	0.2	0.00
94			*Cardiocladius* sp.	0.1	0.00
95			*Chironomus* sp.	1.9	1.22
96			*Cladotanytarsus* sp.	0.1	0.00
97			*Conchapelopia* sp.	0.7	0.21
98			*Cryptochironomus* sp.	0.4	0.17
99			*Cryptotendipes* sp.	0.1	0.04
100			*Dicrotendipes* sp.	0.2	0.00

Table S1. Continued.

Taxon #	Name of order	Name of family	Name of species	Individual biomass	Individual biomass SD
101			*Eukiefferiella* sp.	0.04	0.00
102			*Microtendipes* sp.	0.2	0.06
103			*Orthocladius* sp.	0.3	0.20
104			*Orthocladius* spp.	0.3	0.20
105			Chironomidae gen. sp.	0.6	0.62
106			Chironomidae gen. spp.	0.6	0.62
107			*Parametriocnemus* sp.	0.2	0.05
108			*Polypedilum* sp.	0.3	0.05
109			*Potthastia longimana*	0.5	0.00
110			*Pseudorthocladius* sp.	0.6	0.10
111			*Rheopelopia joganflava*	0.6	0.10
112			*Rheotanytarsus* sp.	0.1	N/A
113			*Stictochironomus* sp.	2.8	1.87
114			*Tanytarsus* sp.	0.2	0.04
115			*Tvetenia* sp.	0.1	0.00
116		Simuliidae	*Simulium* sp.	1.2	0.99
117		Athericidae	*Atrichops morimotoi*	0.9	0.56
118		Stratiomyidae	Stratiomyidae gen. sp.	1.4	0.00
119		Dolichopodidae	Dolichopodidae gen. sp.	0.6	0.21
120	Coleoptera	Hydrophilidae	*Laccobius oscillans*	1.8	0.00

Table S1. Continued.

Taxon #	Name of order	Name of family	Name of species	Individual biomass	Individual biomass SD
121		Elmidae	*Ordobrevia maculata*	0.8	0.07
122			*Zaitzevia nitida*	0.5	0.06
123			*Zaitzeviaria brevis*	0.1	0.00
124			*Elminae* sp.	0.6	0.63
125			*Elminae* spp.	0.6	0.63
126		Psephenidae	*Ectopria opaca opaca*	1.4	0.32
127			*Eubrianax granicollis*	6.4	3.94
128			*Mataeopsephus japonicus*	6.8	4.80
129		Lampyridae	*Luciola cruciata*	11.5	16.05
130	Acari	-	*Acarina*	N/A	N/A

Table S2. Numbers of individuals for each taxon (the first sample replication) shown as the value per quadrat (30 cm × 30 cm area) for each site.

Taxon #	Site #														
	1	2	3	4	5	6	7	8	9	10	11	12	13	14	15
1		2													
2															
3												2			
4															
5	10			16								22			
6															
7															
8	1			20								4			
9			5												
10	1														
11	5	1							4			3			
12				12						833			12		
13			1												
14			1							1					
15										1					
16									1						
17			9		1							1			
18															
19															
20															

Table S2. Continued.

Taxon #	Site #														
	1	2	3	4	5	6	7	8	9	10	11	12	13	14	15
21					6										
22		2		1					1						
23		1													
24					1										
25	1	5		4	2										
26		17													2
27	1														
28															
29															
30		3	1	1									10		
31	27	7			8						1				
32											1				
33											4				
34					10	1		2							
35	2	1						7							
36						1					1				
37															
38	2				2										
39															
40					10										

Table S2. Continued.

Taxon #	Site # 1	2	3	4	5	6	7	8	9	10	11	12	13	14	15
41		4			9	3			1						
42											1				
43								1							
44	1				8			1							
45	1														
46			1												
47				2											
48	1														
49															
50						1									
51														1	
52															
53						4	3								1
54						3					1				2
55		1				1		5	1		3				
56						1									
57											1				
58							1								
59															
60															

Table S2. Continued.

Taxon #	Site # 1	2	3	4	5	6	7	8	9	10	11	12	13	14	15
61	2	1													
62															
63															
64	46	11		2	28			3		4			5		
65					2	15									
66															
67							1								
68															
69															
70	2	8			2	14					6			1	3
71						2									
72	2														
73											1				
74								1							
75	8										1				
76											1				
77											2				
78						1									
79											2				
80															2

Table S2. Continued.

Taxon #	Site # 1	2	3	4	5	6	7	8	9	10	11	12	13	14	15
81							1								
82						1									
83			1						1			1			
84		1											1		
85						9	2								
86								1							
87	3		4												
88															
89															
90															
91							1								
92															
93															
94															
95										351					
96	8	8	2	1	1										
97	3			3	4			1		2					
98															
99				67									1		
100															

Table S2. Continued.

Taxon #	Site # 1	2	3	4	5	6	7	8	9	10	11	12	13	14	15
101															
102					2										
103	2					1	1								
104					20										
105	1			2				2							
106		5			54										
107				5											
108	1	9	3	9	42	1			2	57			3		
109						1									
110						1									
111															
112		1			41					1					
113															
114			12		2					2					
115															
116		8							1		1			1	
117				1					1						
118		1													
119															
120				1											

Table S2. Continued.

Taxon #	Site #														
	1	2	3	4	5	6	7	8	9	10	11	12	13	14	15
121															
122															
123	1		7			16			2						
124		1			2			4	1			2			
125	3														
126	1														
127			1						1						
128															
129			1												
130	9	4			2			1						1	

Table S3. Numbers of individuals for each taxon (the second sample replication) shown as the value per quadrat (30 cm × 30 cm area) for each site.

Taxon #	Site # 1	2	3	4	5	6	7	8	9	10	11	12	13	14	15
1		3													
2									1						
3												1			
4		2				2									
5	9											79			
6		1													
7		1													
8												10	1		
9															
10		4													
11		1		4	37			1	1			1			
12	16									858					
13										1					
14															
15															
16									1						
17					1					2					
18	1														
19													1		
20						1								2	

Table S3. Continued.

Taxon #	Site #														
	1	2	3	4	5	6	7	8	9	10	11	12	13	14	15
21															
22		13		1					1						
23											7				
24															
25		1	1	2					3						
26		32					7								2
27															
28						1									
29	2														
30		9							2				20		
31	4	5			8			3			2				
32											5				
33									1		6				
34								2			1				
35	2	1		1				9	1						
36						1					2			2	
37							1								
38	2				6										
39		1				1		2							
40	1				2										

Table S3. Continued.

Taxon #	Site # 1	2	3	4	5	6	7	8	9	10	11	12	13	14	15
41		75					3		2						
42															
43					1			1							
44								1			1				
45															
46															
47															
48	1			10	1							1			
49									1						
50									1					1	
51		2	1					1							
52							1								
53						1	12								1
54						2					4				
55		2			1			4							
56															
57											1				
58															
59															1
60														1	

Table S3. Continued.

Taxon #	Site # 1	2	3	4	5	6	7	8	9	10	11	12	13	14	15
61	5														
62											1				
63							1								
64	25	17		15	6					5			2		
65					1										
66						4									
67															
68															2
69							1								
70		11				10	5				65				
71						1								1	
72															
73								1			1				
74															
75	12			5											
76											2				
77															
78															
79											1				
80															

Table S3. Continued.

Taxon #	Site # 1	2	3	4	5	6	7	8	9	10	11	12	13	14	15
81															
82	1														
83		1	2	1					1						
84						1									
85							36								
86															
87	4	2		7											
88	1														
89													1		
90		1						1							
91			1						1		1				
92							1								
93		1													
94		1													
95										633					
96	19	11	7	2											
97		6		3				1		3					
98			1												
99															
100													8		

Table S3. Continued.

Taxon #	Site # 1	2	3	4	5	6	7	8	9	10	11	12	13	14	15
101		8													1
102					1										
103	1						1	1						1	
104		3	2	8							2				
105															
106		2		2											
107		3													
108	5	35	1	4	7				1		1	12			
109															
110															
111								1							
112					2				1						
113												1			
114					1							1	1		
115		10									1				
116		81		2		3	2				4		10		1
117		1	2	1	3										
118															
119		1													
120															

Table S3. Continued.

Taxon #	Site #														
	1	2	3	4	5	6	7	8	9	10	11	12	13	14	15
121							2								
122		1													
123		4	5			1									
124			3	6	2		1								
125	8	8										2			
126	1			2											
127				3											
128				2				1							
129															
130	19	22	7	4	12			1			1				
